# An observational study on the technical and tactical performance of fan zhendong in table tennis: analysis of attack perspective and line changes

**DOI:** 10.3389/fbioe.2026.1763704

**Published:** 2026-04-29

**Authors:** Ye Jin, Wenlong Ma

**Affiliations:** 1 School of Physical Education, Shanghai University of Sport, Shanghai, China; 2 Zhonghui Investment Management (Tianjin) Co., Ltd., Tianjin, China

**Keywords:** attack perspective, fan zhendong, line change, table tennis, techniques and tactics

## Abstract

**Introduction:**

To provide technical and tactical training references for the Chinese national table tennis team following recent rule changes, and to offer theoretical support for technical–tactical analysis from a biomechanical perspective.

**Methods:**

A notational analysis based on systematic video observation was conducted to examine Fan Zhendong’s technical and tactical characteristics in initiating attacks within the first four strokes (Attack first vs. Counterattack first), as well as the relationship between scoring outcomes and down-the-line shot selection on the final stroke. Biomechanical characteristics of forehand and backhand attacking techniques performed by Fan Zhendong and his opponents were captured using the ITTF Table Tennis Review system, which consists of 17 high-speed cameras, and were analyzed qualitatively through comparative analysis.

**Results:**

(1) The usage rate and scoring efficiency of attack first within the first four strokes were significantly higher than those of counterattack first (P < 0.05). (2) Both the usage rate and scoring efficiency of down-the-line shots on the final stroke were significantly higher than those without directional change (P < 0.05). (3) The biomechanical structure of Fan Zhendong’s backhand technique was superior to that of his opponents.

**Conclusion:**

(1) His technical style is characterized by strong attack initiation, strong confrontation, and strong rally ability. (2) His backhand technique is compact, with efficient and concentrated force production, forming a highly effective backhand technical system. (3) On the final stroke, he effectively mobilizes opponents through down-the-line shot variation to secure match victory.

## 1 Introduction

Table tennis has long been a dominant sport for China. By the conclusion of the 2024 Paris Olympics, 117 Chinese players had claimed world champion titles, earning a total of 267 gold medals at major international events (Jin, 2025). Since the sport was introduced to the Olympics at the 1988 Seoul Games, ten editions have featured table tennis, producing 29 Chinese Olympic champions (Sohu.com, 2024a). The Chinese team has secured the men’s singles gold medal seven times and the women’s singles title ten times (Tencent.com, 2024a; Tencent.com, 2024b). Of the eleven Grand Slam winners in history, ten are from China, underscoring the country’s unrivaled position in the sport (Sohu.com, 2024b). China has sustained its excellence through a strong commitment to innovation and openness, consistently demonstrating the true spirit of table tennis on the world stage (Wang and Guo, 2022). To enhance spectator appeal, the ITTF introduced a series of rule changes after 2000: in October 2000, the ball diameter increased from 38 mm to 40 mm; in September 2001, the scoring system changed from 21 to 11 points per game; in September 2002, the hidden serve was banned (Wang J., 2014)^.^ After the 2008 Beijing Olympics, organic glue was replaced by non-organic alternatives. On 1 July 2014, celluloid balls were phased out in favor of eco-friendly acetate-based balls (Lingxuan et al., 2023; Cheng, 2014). In 2017, DHS introduced the “D40+” ABS ball for all major events including the World Cup and ITTF World Tour Finals (ITTF, 2025). In June 2017, mixed doubles was added to the Tokyo Olympics program (Sohu.com, 2025a). Changes to ITTF regulations have altered the ball’s material and size, reducing spin during rallies. As a result, most players—especially on the men’s side—tend to initiate attacks earlier, reducing the number of transitional shots. Modern men’s matches emphasize tactical variation; those who adapt first often gain a decisive advantage. With evolving rules and formats, China’s dominance has faced new challenges.

During the Paris Olympic cycle, Fan Zhendong consistently ranked among the world’s top two. As a right-handed shakehand grip player using inverted rubber on both sides, his playing style combines topspin loops with quick attacks. He has achieved multiple titles and runner-up finishes in major international competitions. He captured men’s singles titles at the World Championships in Houston and the WTT Cup Finals in Singapore, and claimed the men’s singles title at the 2022 National Championships and the men’s team title at the Chengdu World Team Championships. He won the men’s singles titles at the 2023 Durban World Championships and the 2024 Paris Olympic Games, completing the career Super Grand Slam (Sohu.com, 2025b). Currently, the core lineup of the Chinese team lacks the stability to anchor the squad, and the contribution of experienced players remains crucial. Although Fan Zhendong has not announced retirement, his consistent performance and refined technique continue to make him a key figure in the men’s team (Tencent.com, 2025a; Tencent.com, 2025b; NetEase, 2025).

In research on table tennis technical–tactical evaluation methods, the three-phase analysis method has been highly influential. However, with the continuous evolution of table tennis techniques and tactics, as well as a series of competition rule modifications implemented by the International Table Tennis Federation (ITTF) since 2000, the limitations of this method have gradually become apparent, prompting extensive scholarly attention and debate. Studies by Zhang (2004), Dong et al. (2005), Zhang et al. (2005), and Tang et al. (2010), Tang and Zhao (2013), among others, have refined and innovated the three-phase analysis method based on a “segmentation” concept. In the field of sports biomechanics, existing work has primarily focused on whole-body kinematic parameters during forehand topspin strokes—such as shoulder horizontal flexion angle, elbow flexion angle, hip flexion/extension, and knee flexion torque (Chen et al., 2022)—and on biomechanical parameters of backhand topspin strokes, including wrist flexion angle, shoulder flexion angular velocity, shoulder abduction angular velocity, and the thorax–pelvis internal rotation angular velocity (Xing et al., 2022). (1) Technical–tactical research has predominantly adopted the three-phase analytical framework, and although some studies have addressed stroke initiative, the tactical impact of directional changes has not been systematically examined. This study, conducted within the framework of the traditional three-stage research model, specifically focuses on the technical and tactical characteristics of “Attack First” and “Counterattack First” strategies during the initial four strokes. Additionally, it examines the utilization of forehand and backhand techniques and their associated scoring outcomes during these phases.

In addition, it innovatively examines the relationship between scoring outcomes and down-the-line shot selection on the final stroke, analyzing the usage and effectiveness of tactics with and without directional change. The findings aim to provide practical training references for the Chinese national table tennis team in the context of recent rule changes. (2) Previous biomechanical studies have been limited to laboratory settings, with no reported analyses conducted under real competition conditions. This study is the first to utilize biomechanical analytical functions derived from technical movements captured by the 17 high-speed cameras of the ITTF Table Tennis Review (TTR) system. A comparative analysis of the biomechanical characteristics of Fan Zhendong and his opponents during forehand and backhand offensive strokes was conducted. A qualitative biomechanical analysis was conducted to explore the reasons behind the high quality and significant scoring effectiveness of Fan Zhendong’s backhand technique, providing a basis for standardizing techniques for young players.

## 2 Research subjects and methods

### 2.1 Research subjects

1. Conduct a quantitative analysis of Fan Zhendong’s tactics of initiating the attack within the first four strokes (early vs. late initiation), including the use of forehand and backhand techniques and their scoring outcomes.
2. Conduct a quantitative analysis of Fan Zhendong’s use of down-the-line tactics on the final stroke, including their frequency and scoring outcomes.
3. Conduct a qualitative analysis of the biomechanical characteristics of Fan Zhendong’s forehand and backhand attacking techniques in matches using the TTR system.

### 2.2 Operational definitions of key terms

The operational definitions of key terms used in this study are presented in Table 1.

**TABLE 1:** Operational definitions of key terms.

Title	Description
Serve-attack phase	First and third strokes
Receive-attack phase	second and fourth strokes
Rally phase	Fifth stroke and beyond
Phase scoring rate	(Points won in phase)/(points won + points lost in phase) × 100%
Phase utilization rate	(Points won + points lost in phase)/(total points won + total points lost in match) × 100%
Direct scoring	A point won immediately after the execution of a specific technical action during a match
Indirect scoring	The final point outcome of the rally won by the player, excluding direct scoring
Direct scoring rate	(Direct scoring ÷ frequency of the corresponding technical action) × 100%
Indirect scoring rate	(Indirect scoring ÷ frequency of the corresponding technical action) × 100%
Line changes	The situations where the direction of the returning player’s shot differs from the direction of the opponent’s incoming ball. This includes three changes: Change to cross-court line, change to down-the-line, and change to center down line
Non-directional changes	The situations where the direction of the returning player’s shot is consistent with the direction of the opponent’s incoming ball. This includes cross-court rally and down-the-line rally
The line divisions	As shown in Figure 1
Attack first	Situations in which fan zhendong initiates the offensive action first within a given tactical phase. Specifically, it includes: (1) situations in which the player is the first to convert backspin into topspin during a rally; (2) situations in which, after the opponent plays a topspin stroke, the player returns the ball using an attacking technique
Counterattack first	Situations in which the opponent initiates the offensive action first, and fan zhendong responds by executing a secondary attacking technique
Unexpected	The ball goes out/into the net/hits the net and goes out in table tennis matches

**FIGURE 1:**
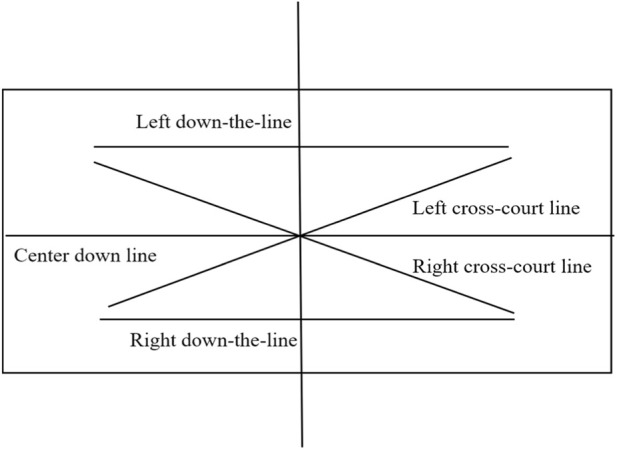
Line classification for right-handed players.

### 2.3 Research methods

#### 2.3.1 Literature review method

In accordance with the research objectives and direction, relevant literature was retrieved using the keywords “table tennis tactics and techniques” and “Fan Zhendong” via the CNKI database, yielding 256 related sources. Further keyword searches using “initiating stroke” and “directional change” produced 45 closely related references, including 10 journal articles, 32 academic theses, and 3 conference papers. To ensure comprehensive and accurate data collection, additional sources were consulted from the Beijing Sport University Library, the National Library of China, and relevant digital resources. These references were organized, analyzed, and synthesized to provide theoretical support for this thesis.

#### 2.3.2 Video observation method

This study selected 45 matches between Fan Zhendong and top offensive players, with 40 matches without the Hawk-Eye system for tactical and technical statistical analysis, and 5 matches with the Hawk-Eye system for biomechanical qualitative analysis.

##### 2.3.2.1 The selection of video footage for technical and tactical analysis

Match footage was collected from publicly available platforms, including Migu Video and Bilibili. A total of 40 matches played by Fan Zhendong between 2021 and 2023 were selected for analysis. The selection criteria included:

1. Priority was given to major tournaments such as the three major world championships, WTT Series, and the Asian Championships.
2. Matches analyzed were primarily finals, semifinals, quarterfinals, and round-of-16 matches.
3. Opponents were mainly top 20 male singles players in world rankings.
4. The sample comprised 12 final matches, 10 semifinal matches, 9 quarterfinal matches, and 9 round-of-16 matches, yielding a total of 40 matches. All selected matches were drawn from top-tier international and national competitions, including the Olympic Games, World Championships, WTT Grand Smash, WTT Champions, WTT Cup Finals, Asian Championships, and National Championships, representing the highest competitive level in men’s table tennis, characterized by strong tactical intensity and stable performance quality (as shown in Table 2).

**TABLE 2:** Statistical overview of fan Zhendong’s 40 matches.

Year	Match	Opponent	Score
2021	Tokyo Olympics Men’s singles semifinal	Lin Yun-Ju	4:3
2021	Tokyo Olympics Men’s singles final	Ma Long	2:4
2021	Tokyo Olympics Men’s team final	Dimitrij Ovtcharov	3:2
2021	Nanyang WTTC trials Men’s singles semifinal	Wang Chuqin	4:2
2021	Shaanxi national games Men’s singles semifinal	Liang Jingkun	4:0
2021	Shaanxi national games Men’s singles final	Liu Dingshuo	4:0
2021	Houston WTTC Men’s singles semifinal	Liang Jingkun	4:1
2021	Houston WTTC Men’s singles final	Truls Möregårdh	4:0
2021	WTT cup Finals Singapore Men’s singles final	Tomokazu Harimoto	4:1
2022	WTT champions Macao quarterfinal	Xu Yingbin	2:3
2022	WTT Singapore Grand smash round of 64	Anton Källberg	3:0
2022	WTT Singapore Grand smash round of 16	Ahn Jae-Hyun	3:2
2022	WTT Singapore Grand smash quarterfinal	Patrick Franziska	3:0
2022	National championships Men’s singles final	Lin Gaoyuan	4:2
2022	Chengdu WTTC Men’s team quarterfinal (CHN vs. SWE)	Mattias Falck	3:0
2022	Chengdu WTTC Men’s team semifinal (CHN vs. JPN)	Shunsuke Togami	3:0
2022	Chengdu WTTC Men’s team semifinal (CHN vs. JPN)	Tomokazu Harimoto	2:3
2022	Chengdu WTTC Men’s team final (CHN vs. GER)	Benedikt Duda	3:0
2022	WTT champions Macao quarterfinal	Darko Jorgić	3:0
2022	WTT champions Macao final	Wang Chuqin	3:4
2022	WTT Cup finals xinxiang quarterfinal	Dimitrij Ovtcharov	2:3
2023	Durban WTTC trials Men’s singles final	Ma Long	0:3
2023	WTT Singapore Grand smash second round	Tiago apolónia	3:0
2023	WTT Singapore Grand smash round of 16	Lin Yun-Ju	3:0
2023	WTT Singapore Grand smash quarterfinal	Truls Möregårdh	4:2
2023	WTT Singapore Grand smash final	Ma Long	4:1
2023	WTT champions xinxiang round of 16	Lee sang-su	3:1
2023	WTT champions xinxiang quarterfinal	Lin shidong	3:2
2023	WTT champions xinxiang semifinal	Lin Yun-Ju	4:3
2023	WTT champions xinxiang final	Liang Jingkun	4:1
2023	WTT champions Macao round of 16	Lee sang-su	3:0
2023	WTT champions Macao quarterfinal	Alexis Lebrun	2:3
2023	Durban WTTC Men’s singles round of 16	Qiu Dang	4:0
2023	Durban WTTC Men’s singles quarterfinal	Omar assar	4:0
2023	Durban WTTC Men’s singles semifinal	Liang Jingkun	4:2
2023	Durban WTTC Men’s singles final	Wang Chuqin	4:2
2023	WTT star contender Goa round of 32	Cho Dae-seong	2:3
2023	WTT Contender Zagreb semifinal	Lin shidong	3:1
2023	Asian championships Men’s singles semifinal (korea)	Liang Jingkun	3:1
2023	Asian championships Men’s singles final (korea)	Ma Long	3:2

##### 2.3.2.2 The selection of video footage for biomechanical qualitative analysis

The TTR system was first used at the 2025 Men’s World Cup. By the end of 2025,it has been applied to five of Fan Zhendong’s matches at the 15th National Games only. The match stage, opponents, and scores are shown Table 3.

**TABLE 3:** Match information of fan Zhendong’s five TTR-Recorded matches at the 15th national games.

Date	Category	Round	Opponent	Score
Nov.10, 2025	MS	1/16	Zhou Yu	4:0
Nov. 15, 2025	MS	1/2	Wang Chuqin	4:2
Nov. 16, 2025	MS	Final	Lin shidong	4:1
Nov. 18, 2025	MT	1/4	Liang Jingkun	3:2
Nov. 20, 2025	MT	Final	Wang Chuqin	3:1

#### 2.3.3 Technical and tactical statistical methods

##### 2.3.3.1 Data collection

Considering that there are many varying factors in each rally of a table tennis match, this study analyzes each rally as the basic unit of analysis. A total of 3,139 rallies were recorded across the 40 selected matches involving Fan Zhendong. Of these, 108 rallies were identified as irregular points, including edge balls, net balls struck by Fan Zhendong, opponent service errors, and points affected by rule violations. In addition, 114 rallies were excluded because the final stroke was not successfully contacted during the rally phase, making it impossible to determine a clear directional trajectory. After removing these invalid observations, 2,917 rallies were retained as valid samples for subsequent statistical analysis.

##### 2.3.3.2 Purpose of video selection

###### 2.3.3.2.1 Video-based statistical analysis

Building on basic statistics obtained using the traditional three-phase analysis method, this study—according to its defined research scope, namely, examining the technical–tactical characteristics of players who initiate first versus respond later in the serve-and-attack phase and the receive-and-attack phase, as well as the variation in shot placement of the final stroke in the rally phase—determined the statistical methods, designed statistical coding forms, and standardized the coding procedures. A standardized statistical protocol was ultimately developed and then subjected to expert evaluation for reliability and validity. To ensure data quality, all video coding in this study was conducted independently by members of the research team. The Kappa consistency test is used to assess the level of agreement between two sets of data (or methods) (Fink, 2014). In fields such as sport performance analysis and notational analysis, the reliability of data collection is commonly evaluated through inter-observer agreement (Abdullah et al., 2016). Assessing inter-observer agreement reflects the reliability of the data recording process and contributes to improving the accuracy and credibility of research findings (Ciuffarella et al., 2013). In this study, two researchers independently recorded the data, after which an agreement analysis was conducted. The results showed a Cohen’s kappa coefficient of 0.916 (p < 0.01) between the two datasets, indicating a high level of agreement. This demonstrates that the data recording method adopted in this study is highly reliable and provides robust evidence for the reliability of match data collection, supporting its continued use in subsequent research. Two researchers performed the coding separately, and any rounds with discrepancies were subsequently reviewed and discussed until a consensus was reached, after which the final results were confirmed and recorded. Recent analyses of high-level athletes’ technical and tactical applications on a round-by-round basis in elite competitions allow opponent-related variables to be controlled within a minimal range. This approach enables a more accurate identification of individual athletes’ technical–tactical tendencies, thereby facilitating the formulation of more precise competition strategies and training programs.

###### 2.3.3.2.2 Expert pedagogical observation

Experts with extensive experience in providing technical–tactical support services were invited to watch the videos. Using a pedagogical observation approach, they provided subjective evaluations of athletes’ competitive performance during matches, including their technical–tactical execution, physical condition, and psychological changes. This procedure was intended to enhance the relative accuracy and scientific rigor of the video-based discussion presented in this paper.

### 2.3.4 Control of experimental conditions

**FIGURE 2:**
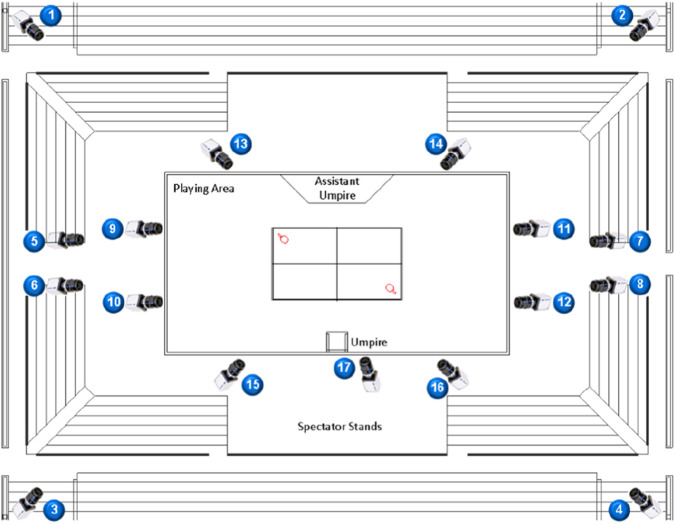
Layout of TTR camera positions.

1. Camera placement: During competition, TTR camera positions were arranged in strict accordance with the relevant regulations. The placement and angles of Camera No. 1 at the 15th National Games are shown in Figure 2.
2. Selection of test actions: To ensure technical consistency across athletes and across repeated strokes, the sampled actions were constrained with respect to the opponent’s incoming ball characteristics at the moment of contact, as well as the player’s positioning, shot direction/placement, and force application when returning the ball.
3. Selection of outcome variables: Qualitative assessments were conducted for the backswing phase, forward swing/impact phase, and follow-through phase, focusing on wrist, elbow, and shoulder joint angles; angular/joint velocities of upper-limb joints; the proximal-to-distal sequencing of force generation in the upper extremity; joint velocities of the lower extremity; center-of-mass displacement; and related indicators.

### 2.3.5 Mathematical and statistical methods

This study employed SPSS software to conduct a one-way analysis of variance (ANOVA). First, the Shapiro–Wilk (S–W) test was used to examine the normality of the sample data. For data that did not conform to a normal distribution, a square root transformation was applied to make the distribution closer to normality. Second, Levene’s test was conducted to assess the homogeneity of variances. Based on the results, appropriate *post hoc* tests were selected. When the assumption of homogeneity of variances was met, the LSD (Least Significant Difference) test was used. When the assumption was violated, Tamhane’s T2 test was applied.

## 3 Result

### 3.1 Overall three-phase technical–tactical analysis

#### 3.1.1 Overall technical–tactical analysis based on the three-phase method

**FIGURE 3:**
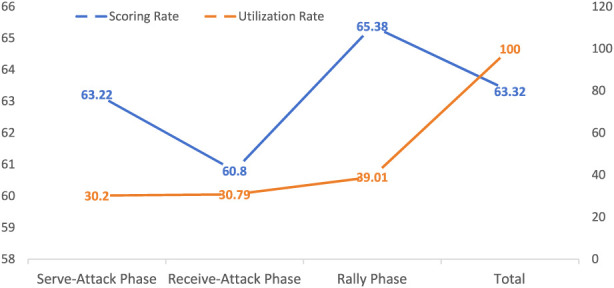
Overall analysis of the three tactical phases.

As shown in Figure 3, Fan Zhendong achieved a scoring rate of over 50% in all three phases, with the highest performance observed in the rally phase. The usage rates in the serve-attack and receive-attack phases were broadly similar, whereas the usage rate in the rally phase was higher than in the other two phases. A statistically significant difference was found between the scoring rate in the rally phase and that in the receive-and-attack phase (p < 0.05). It is demonstrated that Fan Zhendong possesses strong scoring ability in all three tactical phases. The high usage rate and scoring efficiency during the rally phase contribute to maintaining a stable mindset in the attacking and receiving phases, ensuring consistency in his competitive performance.

#### 3.1.2 Technical–tactical analysis of attack first and counterattack first in the first four strokes

**FIGURE 4:**
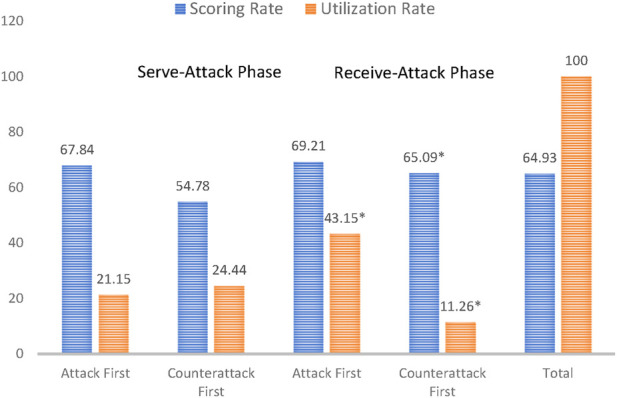
Overall analysis of the initiating stroke performance in the serve-attack and receive-attack phases. Note: * indicates statistical significance between the serve-attack phase and the receive-attack phase with p < 0.05.

As shown in Figure 4, Fan Zhendong has a lower usage rate of forehand techniques in the Serve-Attack Phase compared to the Receive-Attack Phase, with a higher usage rate of backhand techniques in the Serve-Attack Phase. This indicates that both players tend to use forehand techniques more for receiving serves and control techniques for receiving the ball. In both the Serve-Attack and Receive-Attack Phases, the scoring rate for Fan Zhendong’s forehand techniques in the Serve-Attack Phase and backhand techniques in the Receive-Attack Phase is above 65%, suggesting that he can effectively use forehand techniques to score within the first four strokes, and when his opponent attacks first, he can also effectively initiate a counterattack using backhand techniques.

### 3.2 Technical–tactical analysis of forehand and backhand attacking techniques

#### 3.2.1 Technical–tactical analysis of forehand and backhand attacking techniques in different offensive states

**FIGURE 5:**
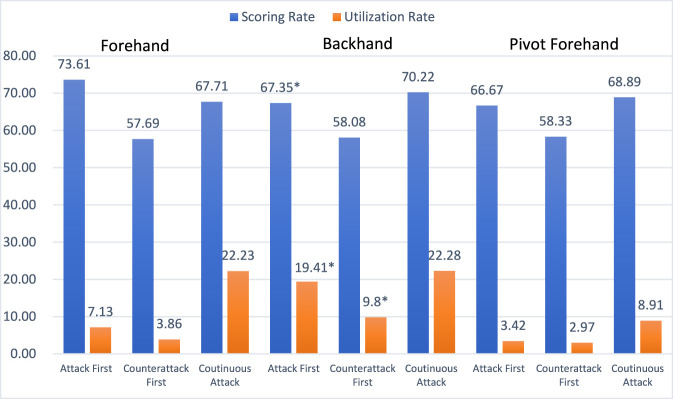
Application of forehand and backhand techniques across different offensive states.

As shown in Figure 5, Fan Zhendong predominantly relies on backhand techniques to execute both attack first and counterattack first, with a usage rate significantly higher than that of the forehand. However, in the final stroke of a continuous offensive sequence, the use of forehand and backhand techniques becomes more balanced. This pattern is consistent with the current technical–tactical trend in men’s table tennis—under the emphasis on strong initiative, strong confrontation, and strong rallying ability—characterized by “creating advantage with the backhand and scoring with the forehand.” Fan Zhendong’s scoring rates exceed 50% across all offensive states, indicating an overall competitive advantage. In contrast, the scoring rate for “counterattack first” is lower than that of the other two offensive states, suggesting that Fan Zhendong’s control ability requires improvement.

#### 3.2.2 Technical–tactical analysis of forehand and backhand attacking techniques in the second stroke

**FIGURE 6:**
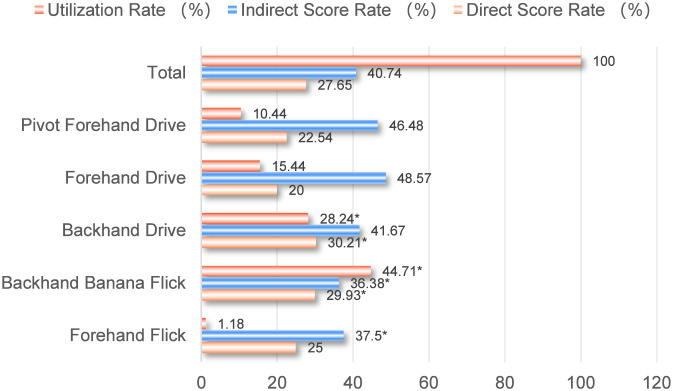
Utilization rate, direct and indirect scoring rate of first-attack techniques in the second stroke.

As shown in Figure 6, when receiving, in the second stroke, Fan Zhendong initiated attacks significantly more often with the backhand than with the forehand. In particular, the usage rate of the backhand banana flick was significantly higher than that of other techniques. Additionally, the direct point-winning rate of backhand attacking techniques was significantly higher than that of forehand attacking techniques, indicating that Fan Zhendong’s backhand technique is of high quality and capable of directly scoring when initiating the attack. However, the indirect point-winning rates did not show the same pattern, suggesting that while the forehand has an advantage in consecutive attacks after a backhand offensive, the ability to maintain consecutive attacks after a backhand offense needs improvement.

#### 3.2.3 Comparison of biomechanical characteristics of forehand and backhand techniques based on the TTR

Using motion analysis derived from the TTR, this study conducted a biomechanical assessment of the forehand and backhand techniques of Fan Zhendong and his opponents. The results indicate that: (1) no significant differences were observed in forehand technique between Fan Zhendong and his opponent; and backhand technique differed significantly between the two players. The comparison of backhand technique is presented in Table 4:

**TABLE 4:** Biomechanical characteristics of fan and his opponent during the backswing phase.

Biomechanical parameter	Wrist joint movement	Elbow joint angle	Shoulder joint angle	Racket position
Fan Zhendong	Smaller	Smaller	Smaller	Slightly above the table
Opponent	Larger	Larger	Larger	Below the table

As shown in Table 4, Fan Zhendong demonstrates a smaller backswing amplitude during the backhand attacking action. The shoulder joint accomplishes the forward elbow thrust primarily through internal rotation, which is accompanied by a greater range of elbow flexion as well as increased wrist flexion and ulnar deviation (adduction). This pattern may facilitate greater energy storage during the backswing phase. Moreover, a higher racket position enables faster and more efficient forward force production.

As shown in Table 5, during backhand attacking actions, Fan Zhendong exhibits a stable center of mass at ball contact, an earlier contact timing, and a contact point closer to the body. In addition, the elbow drives forward while the racket position remains relatively fixed, enabling a combined hit-and-brush contact pattern. Collectively, these characteristics allow Fan Zhendong to express force more effectively at impact, resulting in higher return speed and greater ball power, thereby facilitating direct point scoring or creating additional attacking opportunities for the forehand. If, on this basis, he further increases tactical variability through adjustments in spin, speed, power, and rhythm within his tactical system, his overall competitive performance may be enhanced more effectively.

**TABLE 5:** Biomechanical characteristics of fan and his opponent during the forward swing.

Biomechanical parameter	Elbow joint angle	Elbow range of motion	Shoulder joint angle	Racket–ball contact point	Force application pattern	Timing of contact	Body center of mass
Fan Zhendong	Smaller	Smaller	Smaller	Fixed	Combination of hitting and brushing	Earlier	Stable
Opponent	Larger	Larger	Larger	Variable (not fixed)	Primarily brushing	Later	Fluctuating

As shown in Table 6, after the follow-through in Fan Zhendong’s backhand attacking action, the elbow joint angle is larger, indicating a greater overall elbow excursion throughout the movement. In addition, the predominantly forward-directed elbow drive can effectively enhance return quality, which is consistent with current technical–tactical trends emphasizing strong initiative (early dominance), strong confrontation, and strong rallying ability.

**TABLE 6:** Biomechanical characteristics of fan and his opponent during the follow-through phase.

Biomechanical parameter	Elbow joint angle	Direction of elbow motion
Fan Zhendong	Larger	Predominantly forward
Opponent	Smaller	Forward and upward

### 3.3 Technical–tactical analysis of line change

#### 3.3.1 Technical–tactical analysis of line change on the final stroke

**FIGURE 7:**
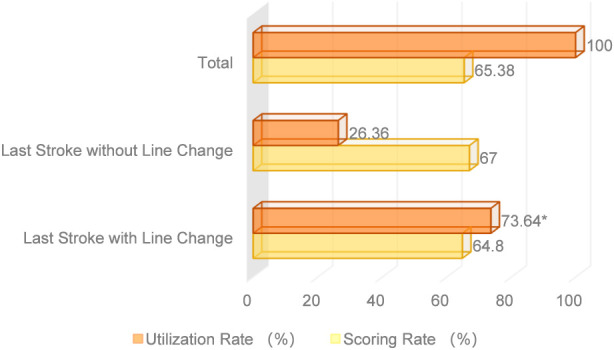
Overall analysis of directional changes vs. non-directional play in the final stroke of the rally phase.

As shown in Figure 7, Fan Zhendong exhibited a high scoring rate on the final stroke regardless of whether a directional change was used. However, the usage rate of directional change on the final stroke was significantly higher than that of no directional change. This indicates that Fan Zhendong is able to actively choose to change direction based on his opponent’s body position and the situation of the return, leading to errors in the opponent’s counterattacks during their movement.

#### 3.3.2 Line-change analysis of the final stroke in the rally phase

**FIGURE 8:**
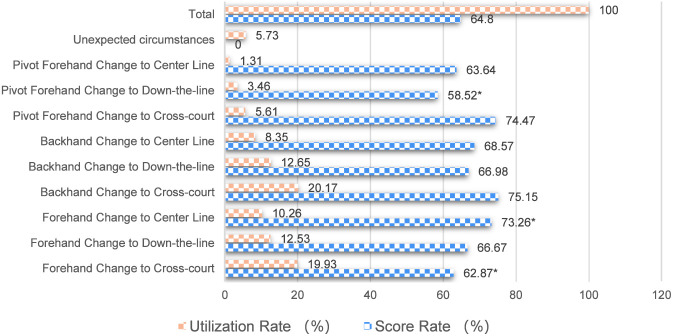
Utilization rate and direct scoring rate of directional changes in the final stroke.

As shown in Figure 8, during the rally phase, the scoring rate for side-on strokes with a cross-court directional change was relatively high, indicating that Fan Zhendong faces issues with positioning during side-on attacks, leading to slightly shorter attack distances and more errors when the center of gravity fails to follow during straight-line shots. The scoring rates for the three directional changes in backhand attacking techniques were all relatively high, suggesting that Fan Zhendong’s backhand attacking technique is stable, allowing him to secure victories through directional changes. The scoring rate for forehand attacking techniques with a cross-court directional change was the lowest, indicating that Fan Zhendong’s forehand is not sufficiently protected during rallies, and when the ball is hit slightly late, he is unable to actively rotate his hips, resulting in errors.

#### 3.3.3 Non-directional changes analysis of the final stroke in the rally phase

**FIGURE 9:**
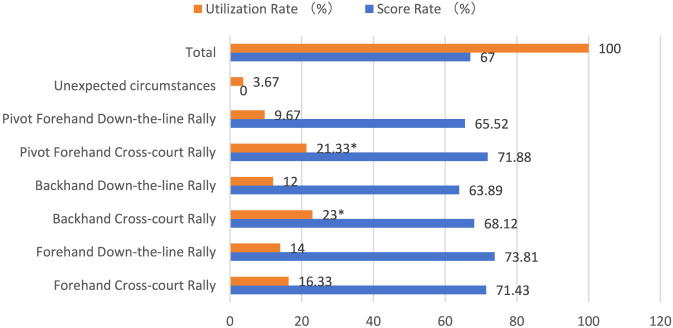
Utilization rate and scoring rate of non-directional changes in the final stroke.

As shown in Figure 9, during the final stroke of the rally phase, for final-stroke attacking actions without a line change, the usage rate of forehand attacks was significantly higher than that of backhand attacks, and the scoring effectiveness was also clearly superior. In particular, the scoring rate for forehand attacks, both down-the-line and cross-court, was higher than 70%, indicating that when Fan Zhendong takes the initiative, he frequently uses his forehand to pressure his opponent, leading to mistakes. In the backhand corner, the usage rate and scoring rate of cross-court pressure were higher than that of down-the-line pressure for both forehand and backhand attacks. This suggests that during even rallies, Fan Zhendong mainly relies on pressure without directional changes, thus avoiding giving his opponent an opportunity to counterattack.

## 4 Discussion

### 4.1 Fan Zhendong’s strong scoring ability in attack first and counterattack first against opponents

Table tennis is a net-separated confrontational ball sport. Studies by Strauss (1987) (Strauss and Arnold, 1987), Hirotsu et al. (2004), and Newton (2009) (Newton and Aslam, 2009) have indicated that in ball games, one player’s competitive behaviors are often significantly influenced by those of the opponent. Under the influence of opponents’ technical and tactical execution, Fan Zhendong demonstrates outstanding scoring efficiency in both attack-first situations and continuous offensive play. This indicates that Fan Zhendong possesses strong offensive awareness and is able to maintain an advantage in attack-first play, counterattack-first situations, and rally exchanges. Even when the opponent attacks first, he is able to effectively reduce the quality of the opponent’s initial attack, thus laying a solid foundation for strong confrontational exchanges. In subsequent continuous offensive phases, he demonstrates powerful attacking capabilities on both the forehand and backhand sides. His offensive techniques are characterized by compact movements, early contact points, and concentrated force application, which are consistent with the technical characteristics and tactical style of elite male players. From a technical standpoint, Fan’s positioning in rallies is reasonable, allowing him to actively adjust his distance from the table based on the opponent’s shot rhythm. However, in the execution of techniques and tactics, excessive emphasis on “strength” may also reduce the scoring rate. Therefore, to improve his winning rate against high-level players in the future, greater emphasis should be placed on incorporating an awareness of tactical variation into his overall tactical system, so as to further enhance scoring efficiency across different technical and tactical phases.

### 4.2 A well-developed backhand system is fan Zhendong’s key technical–tactical weapon for scoring

Fan Zhendong’s backhand techniques (e.g., the backhand banana flick, backhand fast topspin drive, and backhand punch) demonstrate distinctive advantages in their force-generation mechanisms. The qualitative biomechanical analysis indicates that his backhand strokes are characterized by compact motion, concentrated force application, and rapid recovery. These features not only help maintain stability of the body’s center of mass but also effectively reduce unnecessary movement, thereby improving shot accuracy and continuity. Multiple studies have also shown that backhand techniques conform to the principle of sequential joint activation, which enables the racket to achieve higher velocity (Xiao and Wu, 2016; Wang L. L. Y., 2014; Zhang, 2015). In the receiving phase, he demonstrates a high level of attacking awareness and initiative, particularly with his backhand flick, which has become a trademark weapon against short serves. This technique not only generates speed and spin but also effectively disguises the shot, making it difficult for opponents to anticipate, thus providing him with the opportunity to launch an offensive. This finding is consistent with previous studies, which have identified the banana flick as a key weapon among elite male players in the post-40+ ball era, especially under the shortened rally structure (Yu, 2023; Zhou, 2022). Fan Zhendong’s backhand attacking technique has high shot quality, allowing him to take the initiative in confrontations with his opponents. However, the excessive use of the backhand also leads to a decrease in his scoring rate during extended rallies. Therefore, forehand and backhand techniques should be selected based on the opponent’s playing style and technical–tactical strengths, so as to maximize the advantages of the backhand system and its capacity to create opportunities for the forehand.

### 4.3 Line change and attack-defense in rally phases

The rally phase is the core segment in assessing an athlete’s overall technical and tactical ability. Most previous studies agree that, in competition, world-class athletes gain a greater advantage by proactively and flexibly initiating line changes than by adopting an overly conservative strategy that avoids changing lines (Zhang and Xu, 2017; Guo et al., 2018). Fan Zhendong demonstrates exceptional ability in attack-defense transitions and rhythm control during this phase. He not only maintains initiative in the rally but also flexibly adjusts his line choices based on the opponent’s positioning and shot quality. For example, during backhand exchanges, if Fan Zhendong observes that a right-handed opponent is slow to recover position after moving to strike the ball, he may proactively change down the line, exploiting the shorter trajectory and using speed and power to score. When rallies reach a stalemate, he may vary the spin intensity to disrupt rhythm, induce errors, and then switch cross-court to attack the wide angles. Against a left-handed opponent in backhand down-the-line exchanges, he may suddenly target the middle to reduce the opponent’s return quality before shifting cross-court to gain the advantage. In particular, when handling consecutive middle-line shots from the opponent, he often breaks the deadlock by changing the direction of his attacks, achieving points through tactical rhythm shifts. In multi-shot exchanges, he focuses on using angle variation to widen the opponent’s recovery path, thus weakening their ability to sustain long rallies. However, research has shown that in continuous line changes, when facing opponents with strong defensive adaptability, Fan’s choice of final shot direction can become predictable, allowing opponents to successfully defend or counterattack. Furthermore, in high-intensity rallies, his transition from forehand to backhand sometimes leads to slight decreases in stroke stability due to the combined pressures of time and space, resulting in fluctuations in shot quality.

### 4.4 Implications of this study for the training of young table tennis players

1. While emphasizing the quality of returns in the process of developing “strong first attack,” “strong confrontation,” and “strong rallying,” greater emphasis should also be placed on increasing variations in return speed, power, rhythm, and placement. This approach would increase the difficulty for opponents to adapt, reduce direct errors, and effectively enhance his indirect scoring ability.
2. To improve the integrity of his receiving system, future training should focus on counteracting various types of serves and simulate high-pressure competition environments to enhance his judgment and execution in complex spin and rhythm changes. Additionally, his line selection after receiving should become more diverse and unpredictable, avoiding the risk of his tactical patterns being anticipated by opponents. By strengthening his abilities in line variation, timing, and placement control, he can enhance the overall effectiveness and tactical sustainability of his receiving attack.
3. In future training, it is essential to further enhance his Line Change strategy and rhythm-changing ability in rallies, as well as improve his countermeasures against opponents’ multi-shot adjustments. Additional high-intensity rally simulation training will help improve his technical stability and tactical execution accuracy under extreme conditions. Attention should also be given to the risk management of specific line changes and repeated training on how to counteract in disadvantaged rally situations, thereby enhancing his tactical countermeasures and achieving a attack-defense approach.

### 4.5 Limitations of the research and future research directions

Although this study has comprehensively analyzed Fan Zhendong’s technical and tactical features through systematic data collection and analysis, several limitations exist. First, the research sample mainly consists of video materials from official competitions and does not encompass the technical details of his training sessions, which may limit the understanding of his tactical structure. Secondly, this study did not incorporate complex variables such as the opponent’s playing style, match score stage, or psychological factors, making it difficult to fully explore Fan’s strategic choices against different opponents and situations. For example, if the opponent has a weak ability to initiate the attack first but a strong ability to counterattack after the opponent’s first move, Fan Zhendong should increase the frequency and improve the quality of control play when no good opportunity to initiate the attack is available. Conversely, he should increase the frequency of initiating the attack when appropriate, so as to ensure an advantage in the receive phase. Additionally, this study focuses on a single elite athlete, which inherently limits the generalizability of the findings. While a single-case analysis can offer detailed insights, the conclusions drawn from this study may not be directly applicable to other athletes or broader training and competition contexts. Hence, the scope of inference should be more carefully defined, particularly when considering different playing styles or competitive environments. Future research could integrate multi-dimensional data analysis methods, including match simulation, real-time physiological load monitoring, and artificial intelligence video analysis, to more finely analyze athletes’ behavior patterns and tactical decision-making mechanisms in specific environments. It is also recommended to expand the sample pool to include top international players with various playing styles for cross-comparison, systematically evaluating Fan Zhendong’s tactical adaptability and evolution trends. This could provide theoretical support for the national team’s individualized training in future preparation cycles. Expanding the sample to include top international players with diverse playing styles would also allow for cross-comparison, enabling a more comprehensive evaluation of Fan Zhendong’s tactical adaptability. This study employed the TTR System to explore the biomechanical characteristics of athletes’ forehand and backhand attacking techniques. However, the limited number of matches in which Fan Zhendong used the TTR system constrains the analysis. In real competition, variations in ball speed, spin, and placement result in corresponding changes in stance, backswing, and contact position for each stroke. Moreover, players cannot wear testing devices during matches, which reduces the precision of quantitative measurements. Therefore, this study conducted only exploratory qualitative analyses. Further validation and methodological refinement are required for accurate quantitative investigation in future research.

## 5 Conclusion

1. Fan Zhendong demonstrates strong scoring ability with both Attack First and Counterattack First techniques in matches, and should focus on enhancing his awareness of tactical variation in the future.
2. Fan Zhendong’s backhand technique is compact and focused, improving his ability to score both directly and indirectly. In the future, he should pay attention to more rationally utilizing both forehand and backhand techniques based on the opponent’s ball trajectory.
3. Fan Zhendong is able to use directional change tactics to improve his scoring ability during rallies. In the future, he should focus on better judging the opponent’s ball placement and strengthening the protection of his forehand for wide-angle shots.

## Data Availability

The original contributions presented in the study are included in the article/Supplementary Material, further inquiries can be directed to the corresponding author.

## References

[B1] Abdullah M. R. Musa R. M. Maliki A. B. H. M. Kosni N. A. Suppiah P. K. (2016). Development of tablet application-based notational analysis system and the establishment of its reliability in soccer. J. Phys. Educ. Sport 16 (150), 951–956. 10.7752/jpes.2016.03150

[B2] Chen M. Z. Wang X. Chen Q. Ma Y. Malagoli Lanzoni I. Lam W. K. (2022). An analysis of whole-body kinematics, muscle strength and activity during cross-step topspin among table tennis players. Int. J. Perform. Analysis Sport 22 (1), 16–28. 10.1080/24748668.2022.2025712

[B3] Cheng B. (2014). Preliminary study on the characteristics of the new seamless plastic table tennis ball and its impact on technical and tactical development. China Sports Sci. Technol. 50 (05), 68–72. 10.16470/j.csst.2014.05.012

[B4] Ciuffarella A. Russo L. Masedu F. Valenti M. Izzo R. E. De Angelis M. (2013). Notational analysis of the volleyball serve. Timişoara Phys. Educ. Rehabilitation J. 6 (11), 29–35. 10.2478/tperj-2013-0013

[B5] Dong Y. Zhu F. J. Gu J. P. (2005). Technical and tactical analysis of the men’s doubles semifinal and final (Süss/Boll) at the 48th world table tennis championships. J. Chengdu Sport Univ. (4), 73–75.

[B6] Fink A. (2014). How to conduct a literature review (ruhe zuohao wenxian zongshu). Chongqing: Chongqing University Press.

[B7] Guo W. X. Zhao L. N. Xu J. W. Liang M. F. (2018). Characteristics and effects of line-change strategy application in elite men’s singles table tennis matches. J. Beijing Sport Univ. 41 (2), 115–120.

[B8] Hirotsu N. Miyaji C. Ezaki N. (2004). “A model for finding optimal tactics in volleyball using markov decision processes,” in The engineering of sport 5. Editors Hubbard M. Mehta R. D. Pallis J. M. (International Sports Engineering Association), 2, 186–196.

[B10] ITTF (2025). ABS table tennis balls - introducing the latest world-class competition ball. Available online at: https://cn.ittf.com/2017/03/absball/ (Accessed January 20, 2026).

[B11] Jin D. (2025). Table tennis: a micro key to open the study of contemporary Chinese history. Hist. Rev. (01), 221.

[B12] Lingxuan K. Ning D. Wu F. (2023). Preparing for the 2024 paris olympics: analysis of technical and tactical skills of world-class athletes in the era of ABS plastic table tennis balls. Sports Sci. 44 (02), 100–109.

[B13] NetEase (2025). How will the main force of Chinese table tennis change in 2025? Intense competition between two, while eight players remain steadily positioned. Available online at: https://www.163.com/dy/article/JJVK16SD0552RWRJ.html (Accessed January 20, 2026).

[B14] Newton P. K. Aslam K. (2009). Monte carlo tennis: a stochastic markov chain model. J. Quantitative Analysis Sports 5 (3), 1–42. 10.2202/1559-0410.1169

[B15] Sohu.com (2024a). The tenth olympic games witness the birth of 29 Chinese table tennis champions. Available online at: https://www.sohu.com/a/797384935_121455647 (Accessed January 20, 2026).

[B16] Sohu.com (2024b). The 11th grand slam winner in table tennis is born! old waldner becomes the greatest in history, zhang yining and Ma long achieve double grand slam. Available online at: https://www.sohu.com/a/798534717_120499963 (Accessed January 20, 2026).

[B17] Sohu.com (2025a). Introduction to the Tokyo olympic table tennis events: mixed doubles introduced for the first time. Available online at: https://www.sohu.com/a/361351782_114977 (Accessed January 20, 2026).

[B18] Sohu.com (2025b). Fan zhendong becomes the first super grand slam winner, rightfully the number one in table tennis. Available online at: https://www.sohu.com/a/892352255_121481603 (Accessed January 20, 2026).

[B19] Strauss D. Arnold B. C. (1987). The rating of players in racquetball tournaments. J. R. Stat. Soc. Ser. C Appl. Statistics 36 (2), 163–173. 10.2307/2347548

[B20] Tang J. Zhao X. (2013). Composition and analysis of match-winning tactical patterns for different offensive playing styles in table tennis. J. Beijing Sport Univ. 36 (3), 123–127.

[B21] Tang J. Cao H. Deng Y. (2010). Composition and application of tactical combination patterns in table tennis matches. J. Beijing Sport Univ. 33 (11), 108–110.

[B22] Tencent.com (2024a). Olympic table tennis men's singles champions: new addition of fan zhendong, increasing China's olympic gold medals to 35. Available online at: https://news.qq.com/rain/a/20240804A06JEK00 (Accessed January 20, 2026).

[B23] Tencent.com (2024b). Chinese women's singles achieve 10 consecutive olympic wins, table tennis champions never lost after the olympics. Available online at: https://news.qq.com/rain/a/20240803A08AZ500 (Accessed January 20, 2026).

[B24] Tencent.com (2025a). World cup collapse the sixth time in the new century that the Chinese men's team lost the championship. Reveal. Signif. Hidden Issues. Available online at: https://news.qq.com/rain/a/20250420A06PEM00 (Accessed January 20, 2026).

[B25] Tencent.com (2025b). Fan zhendong announces withdrawal from world rankings, will continue competing in the 15th national games. Available online at: https://news.qq.com/rain/a/20241227A069HU00 (Accessed January 20, 2026).

[B26] Wang J. (2014). Research on the development of the world table tennis championships. Sports Cult. Guide (11), 80–83.

[B27] Wang L. L. Y. (2014). Biomechanical analysis of table tennis backhand topspin. J. Sports Sci. Med. 13 (2), 372–379.

[B28] Wang H. Guo G. (2022). On the cultural connotation and contemporary value of the ping pong spirit. Adv. Sports Sci. 10 (1), 105–115.

[B29] Xiao D. D. Wu J. P. (2016). A comparative study of the kinematic characteristics of three in-table offensive techniques using the shakehand grip in table tennis. J. Tianjin Univ. Sport 31 (6), 515–518.

[B30] Xing K. Hang L. Lu Z. Mao C. Kang D. Yang C. (2022). Biomechanical comparison between down-the-line and cross-court topspin backhand in competitive table tennis. Int. Journal Environmental Research Public Health 19 (9), 5146. 10.3390/ijerph19095146 35564541 PMC9102447

[B31] Yu J. (2023). Effects of technical-tactical skills on enhancing scoring efficiency when competing with opponents using different handedness in table tennis matchups. Heliyon 9 (2), e13307. 10.1016/j.heliyon.2023.e13307 36816307 PMC9932731

[B32] Zhang X. P. (2004). Quantitative diagnostic methods and practical effectiveness of the tactical training level of the Chinese national table tennis team. Beijing, China: Beijing Sport University.

[B33] Zhang L. L. Y. Z. (2015). Biomechanical analysis of table tennis stroke: a review. J. Sports Sci. Med. 14 (3), 411–422.

[B34] Zhang S. Q. Xu J. W. (2017). Line-change characteristics, effects, and developmental trends in table tennis matches from the perspective of “either straight or diagonal” play. J. Hebei Inst. Phys. Educ. 31 (6), 81–86.

[B35] Zhang X. T. Dou H. Y. Fu Y. Z. (2005). Technical diagnosis and effectiveness evaluation of wang Hao’s key techniques in the men’s team event at the 47th world table tennis championships. J. Shenyang Sport Univ. (1), 104–107.

[B37] Zhou X. (2022). Explanation and verification of the rules of attack in table tennis tactics. BMC Sports Sci. Med. Rehabilitation 14 (1), 6. 10.1186/s13102-022-00396-3 34998429 PMC8742926

