# Supplementary material for: An observational study on the technical and tactical performance of fan zhendong in table tennis: analysis of attack perspective and line changes
Source: Front Bioeng Biotechnol. 2026 Apr 29;14:1763704. doi: 10.3389/fbioe.2026.1763704 (PMC13168176; doi:10.3389/fbioe.2026.1763704)
Supplement: Supplementary file 1 [file Table1.docx]

Table 1. Operational Definitions of Key Terms

| Title | Description |
| --- | --- |
| Serve-attack phase | first and third strokes |
| Receive-attack phase | second and fourth strokes |
| Rally phase | fifth stroke and beyond |
| Phase Scoring Rate | (Points Won in Phase) / (Points Won + Points Lost in Phase) × 100% |
| Phase Utilization Rate | (Points Won + Points Lost in Phase) / (Total Points Won + Total Points Lost in Match) × 100% |
| Direct scoring | a point won immediately after the execution of a specific technical action during a match |
| Indirect scoring | the final point outcome of the rally won by the player, excluding direct scoring |
| Direct scoring rate | (Direct scoring ÷ Frequency of the corresponding technical action) × 100% |
| Indirect scoring rate | (Indirect scoring ÷ Frequency of the corresponding technical action) × 100% |
| Line changes | the situations where the direction of the returning player's shot differs from the direction of the opponent's incoming ball. This includes three changes: change to cross-court line, change to down-the-line, and change to center down line |
| Non-directional changes | the situations where the direction of the returning player's shot is consistent with the direction of the opponent's incoming ball. This includes Cross-court rally and Down-the-line rally |
| The line divisions | As shown in Figure 1 |
| Attack first | situations in which Fan Zhendong initiates the offensive action first within a given tactical phase. Specifically, it includes:(1) situations in which the player is the first to convert backspin into topspin during a rally;(2) situations in which, after the opponent plays a topspin stroke, the player returns the ball using an attacking technique. |
| Counterattack first | situations in which the opponent initiates the offensive action first, and Fan Zhendong responds by executing a secondary attacking technique. |
| Unexpected | the Ball goes out/into the net/hits the net and goes out in table tennis matches |

Table 2. Statistical Overview of Fan Zhendong’s 40 Matches

| Year | Match | Opponent | Score |
| --- | --- | --- | --- |
| 2021 | Tokyo Olympics Men's Singles Semifinal | Lin Yun-Ju | 4:3 |
| 2021 | Tokyo Olympics Men's Singles Final | Ma Long | 2:4 |
| 2021 | Tokyo Olympics Men's Team Final | Dimitrij Ovtcharov | 3:2 |
| 2021 | Nanyang WTTC Trials Men's Singles Semifinal | Wang Chuqin | 4:2 |
| 2021 | Shaanxi National Games Men's Singles Semifinal | Liang Jingkun | 4:0 |
| 2021 | Shaanxi National Games Men's Singles Final | Liu Dingshuo | 4:0 |
| 2021 | Houston WTTC Men's Singles Semifinal | Liang Jingkun | 4:1 |
| 2021 | Houston WTTC Men's Singles Final | Truls Möregårdh | 4:0 |
| 2021 | WTT Cup Finals Singapore Men's Singles Final | Tomokazu Harimoto | 4:1 |
| 2022 | WTT Champions Macao Quarterfinal | Xu Yingbin | 2:3 |
| 2022 | WTT Singapore Grand Smash Round of 64 | Anton Källberg | 3:0 |
| 2022 | WTT Singapore Grand Smash Round of 16 | Ahn Jae-Hyun | 3:2 |
| 2022 | WTT Singapore Grand Smash Quarterfinal | Patrick Franziska | 3:0 |
| 2022 | National Championships Men's Singles Final | Lin Gaoyuan | 4:2 |
| 2022 | Chengdu WTTC Men's Team Quarterfinal (CHN vs SWE) | Mattias Falck | 3:0 |
| 2022 | Chengdu WTTC Men's Team Semifinal (CHN vs JPN) | Shunsuke Togami | 3:0 |
| 2022 | Chengdu WTTC Men's Team Semifinal (CHN vs JPN) | Tomokazu Harimoto | 2:3 |
| 2022 | Chengdu WTTC Men's Team Final (CHN vs GER) | Benedikt Duda | 3:0 |
| 2022 | WTT Champions Macao Quarterfinal | Darko Jorgić | 3:0 |
| 2022 | WTT Champions Macao Final | Wang Chuqin | 3:4 |
| 2022 | WTT Cup Finals Xinxiang Quarterfinal | Dimitrij Ovtcharov | 2:3 |
| 2023 | Durban WTTC Trials Men's Singles Final | Ma Long | 0:3 |
| 2023 | WTT Singapore Grand Smash Second Round | Tiago Apolónia | 3:0 |
| 2023 | WTT Singapore Grand Smash Round of 16 | Lin Yun-Ju | 3:0 |
| 2023 | WTT Singapore Grand Smash Quarterfinal | Truls Möregårdh | 4:2 |
| 2023 | WTT Singapore Grand Smash Final | Ma Long | 4:1 |
| 2023 | WTT Champions Xinxiang Round of 16 | Lee Sang-Su | 3:1 |
| 2023 | WTT Champions Xinxiang Quarterfinal | Lin Shidong | 3:2 |
| 2023 | WTT Champions Xinxiang Semifinal | Lin Yun-Ju | 4:3 |
| 2023 | WTT Champions Xinxiang Final | Liang Jingkun | 4:1 |
| 2023 | WTT Champions Macao Round of 16 | Lee Sang-Su | 3:0 |
| 2023 | WTT Champions Macao Quarterfinal | Alexis Lebrun | 2:3 |
| 2023 | Durban WTTC Men's Singles Round of 16 | Qiu Dang | 4:0 |
| 2023 | Durban WTTC Men's Singles Quarterfinal | Omar Assar | 4:0 |
| 2023 | Durban WTTC Men's Singles Semifinal | Liang Jingkun | 4:2 |
| 2023 | Durban WTTC Men's Singles Final | Wang Chuqin | 4:2 |
| 2023 | WTT Star Contender Goa Round of 32 | Cho Dae-Seong | 2:3 |
| 2023 | WTT Contender Zagreb Semifinal | Lin Shidong | 3:1 |
| 2023 | Asian Championships Men's Singles Semifinal (Korea) | Liang Jingkun | 3:1 |
| 2023 | Asian Championships Men's Singles Final (Korea) | Ma Long | 3:2 |

Table 3. Match Information of Fan Zhendong’s Five TTR-Recorded Matches at the 15th National Games

| Date | Category | Round | Opponent | Score |
| --- | --- | --- | --- | --- |
| Nov.10, 2025 | MS | 1/16 | Zhou Yu | 4:0 |
| Nov. 15, 2025 | MS | 1/2 | Wang Chuqin | 4:2 |
| Nov. 16, 2025 | MS | Final | Lin Shidong | 4:1 |
| Nov. 18, 2025 | MT | 1/4 | Liang Jingkun | 3:2 |
| Nov. 20, 2025 | MT | Final | Wang Chuqin | 3:1 |

Table 4 Biomechanical Characteristics of Fan and His Opponent During the Backswing Phase

|  | Wrist joint movement | Elbow joint angle | Shoulder joint angle | Racket position |
| --- | --- | --- | --- | --- |
| Fan Zhendong | Smaller | Smaller | Smaller | Slightly above the table |
| Opponent | Larger | Larger | Larger | Below the table |

Table 5 Biomechanical Characteristics of Fan and His Opponent During the Forward Swing

|  | Elbow joint angle | Elbow range of motion | Shoulder joint angle | Racket–ball contact point | Force application pattern | Timing of contact | Body center of mass |
| --- | --- | --- | --- | --- | --- | --- | --- |
| Fan Zhendong | Smaller | Smaller | Smaller | Fixed | Combination of hitting and brushing | Earlier | Stable |
| Opponent | Larger | Larger | Larger | Variable (not fixed) | Primarily brushing | Later | Fluctuating |

Table 6 Biomechanical Characteristics of Fan and His Opponent During the Follow-Through Phase

|  | Elbow joint angle | Direction of elbow motion |
| --- | --- | --- |
| Fan Zhendong | Larger | Predominantly forward |
| Opponent | Smaller | Forward and upward |
